# Retinal Lateral Inhibition Provides the Biological Basis of Long-Range Spatial Induction

**DOI:** 10.1371/journal.pone.0168963

**Published:** 2016-12-28

**Authors:** Jihyun Yeonan-Kim, Marcelo Bertalmío

**Affiliations:** Departament de Tecnologies de la Informació i les Comunicacions, Universitat Pompeu Fabra, Barcelona, Spain; Universidade Federal do ABC, BRAZIL

## Abstract

Retinal lateral inhibition is one of the conventional efficient coding mechanisms in the visual system that is produced by interneurons that pool signals over a neighborhood of presynaptic feedforward cells and send inhibitory signals back to them. Thus, the receptive-field (RF) of a retinal ganglion cell has a center-surround receptive-field (RF) profile that is classically represented as a difference-of-Gaussian (DOG) adequate for efficient spatial contrast coding. The DOG RF profile has been attributed to produce the psychophysical phenomena of brightness induction, in which the perceived brightness of an object is affected by that of its vicinity, either shifting away from it (brightness contrast) or becoming more similar to it (brightness assimilation) depending on the size of the surfaces surrounding the object. While brightness contrast can be modeled using a DOG with a narrow surround, brightness assimilation requires a wide suppressive surround. Early retinal studies determined that the suppressive surround of a retinal ganglion cell is narrow (< 100–300 μm; ‘classic RF’), which led researchers to postulate that brightness assimilation must originate at some post-retinal, possibly cortical, stage where long-range interactions are feasible. However, more recent studies have reported that the retinal interneurons also exhibit a spatially wide component (> 500–1000 μm). In the current study, we examine the effect of this wide interneuron RF component in two biophysical retinal models and show that for both of the retinal models it explains the long-range effect evidenced in simultaneous brightness induction phenomena and that the spatial extent of this long-range effect of the retinal model responses matches that of perceptual data. These results suggest that the retinal lateral inhibition mechanism alone can regulate local as well as long-range spatial induction through the narrow and wide RF components of retinal interneurons, arguing against the existing view that spatial induction is operated by two separate local vs. long-range mechanisms.

## Introduction

**Fig 1:**
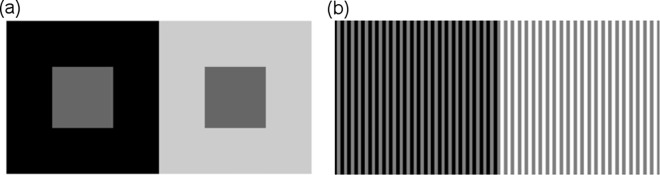
Examples of brightness induction. (a) Brightness contrast: the grey squares have the same luminance, but the one over a black background appears lighter, and the other appears darker. (b) Brightness assimilation: all the grey bars have the same luminance, but the ones over a black background appear darker, while the ones over white appear lighter.

The human visual system works in many ways to reduce the inherent redundancy of visual information in natural scenes, coding it in an efficient way [1–3]. A key mechanism for efficient representation is that of lateral inhibition, which allows to encode the difference in activity between a cell and its surround: in this manner, large homogeneous areas in the scene generate little neural activity and can be represented with less resources. This center-surround organization has important implications for perception as well [4–10], explaining phenomena like simultaneous brightness induction, by which the perceived brightness of an object depends on its surround. Induction can take the form of *contrast*, when the brightness of the object drifts away from that of its neighborhood, e.g. a dark object on a light background appears even darker, or a light object in a dark surround becomes even lighter (see Fig 1A). The reverse is called *assimilation*, in which case the brightness of the objects becomes more similar to that of its surround (see Fig 1B). Whether one perceives assimilation or contrast systematically depends on the size of the surface that surrounds the induction target, such that a large surrounding surface induces contrast on the target while reducing the surrounding size decreases the magnitude of the contrast and ultimately induces assimilation [11–13].

**Fig 2:**
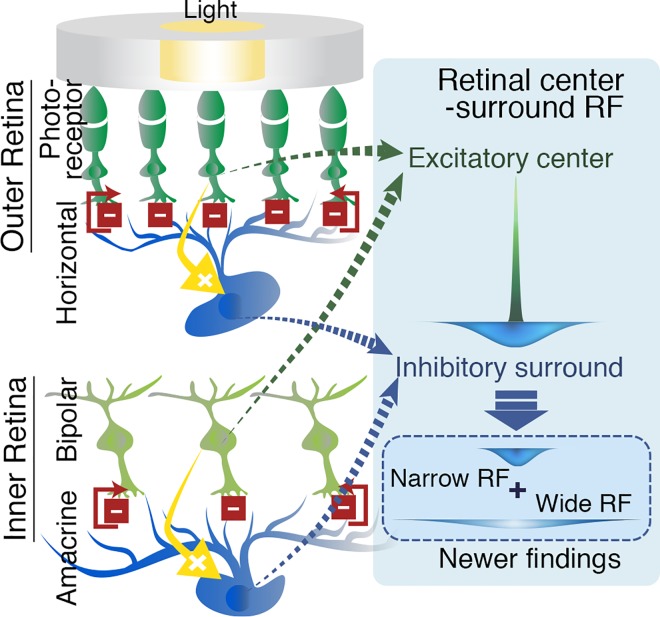
Lateral inhibition process in the retina and the formation of the center-surround RF. Lateral inhibition in the retina occurs as the feedback from the interneurons, horizontal cells and amacrine cells, which receive excitatory inputs from photoreceptors and bipolar cells, respectively, inhibit the excited photoreceptors and bipolar cells and their neighborhood. The typical center-surround RF structure of a ganglion cell is the combination of the excitatory center created by the feedforward cells and inhibitory surround formed by the interneurons. The current study tests the effect of the wide RF component of these interneurons in addition to the classic (narrow) RF (inset).

Lateral inhibition is produced in the retina by interneurons (horizontal and amacrine cells) that pool signals over a neighborhood of presynaptic feedforward cells (photoreceptors and bipolar cells) and send inhibitory signals back to them [14–17] (Fig 2). The typical center-surround RF structure of a ganglion cell is the combination of the excitatory center created by the feedforward cells and the inhibitory surround formed by the interneurons, and is usually modeled as a DOG [5–8,10,15,18,19] in which the standard deviations of the center and the surround Gaussian functions reflect the RF sizes of the feedforward cells and the interneurons, respectively.

Previous studies showed that the DOG computation accounts for the brightness contrast (e.g. [5,7,8,20]). The spatial properties of DOG in these studies are based on the known spatial extent of the ganglion cell RF found in the earlier neurophysiology studies, which is now commonly referred to as ‘the classic RF’ [5–8,10,15,18,19]. The spatial extent of the classic RF is assumed to be narrow, with the surround component ranging from 100–300 μm [15,18,21–23].

On the other hand, this implies that the classic RF of a ganglion cell has a spatial extent much smaller than what would be necessary to generate a brightness assimilation effect because the range of spatial extent that generates a distinguishable assimilation effect much exceeded the classic RF of a ganglion cell [5,12,13,20,24]. For this reason, in the literature there appears to be a consensus that induction cannot be explained by retinal lateral inhibition alone and should involve post-retinal processing. For instance, Reid and Shapley [25] argued that lateral inhibition in the retina generates brightness contrast between a pair of adjacent surfaces while brightness assimilation is generated by a long-range surface interaction operated by an unknown post-retinal neural source, so contrast and assimilation would stem from two independent mechanisms (we call this dual-mechanism hypothesis). Heinemann and Chase [5] supported this claim by computationally showing that lateral inhibition by the classic RF alone cannot explain assimilation, and the full range of the surrounding-size effect in the assimilation-to-contrast phenomena required an additional spatially-global computation process.

However, increasing number of recent neurophysiological studies report that the retinal interneurons exhibit spatially much extended RF profiles (wide RF; > 500–1000 μm; see blue dashed box in Fig 2) [26–31] and that an extra-classical surround component is detected in the ganglion cell responses [21,32–35]. This evidence provides another perspective on the spatial structure of the classic RF that so far has only considered the prominent narrow RF-surround component (< 100–300 μm) [15,18,21–23,36] and suggests that there exists a much wider lateral interaction in the retina.

Here, we computationally demonstrate that the wide RF component of retinal interneurons could generate the kind of long-range surface interaction that is required to produce brightness assimilation. For this we use two biophysical retinal circuitry models that aim to emulate biological voltage responses of the real retinal cells [36–38], models which were designed based on neurophysiological evidence and validated by matching single cell recording data. We find that only when we incorporate the wide RF of the interneurons into these models they become capable of producing results which are compatible with the classic psychophysical data on the surrounding size effect on brightness induction reported by Helson [11] and by Reid and Shapley [12]; also, the spatial extent of the influence of the wide RF component on brightness induction extends up to several degrees, a figure comparable to the psychophysical data by Rudd and Zemach [39]. This suggests the possibility that both brightness contrast and assimilation is produced at the retina by a single mechanism, lateral inhibition.

The current study proposes an unconventional methodology of utilizing biophysical models to predict perceptual data (see also [36]). The data prediction becomes challenging this way, since all the model architectures and parameters are predetermined in the original studies through neurophysiological validation [36–38] and there is no parameter tuning allowed to fit the model output to the perceptual data in our simulations. Nevertheless, the assimilation-to-contrast phenomenon is predicted across the two different models we tested, substantiating the biological justification of our claim.

## Methods: Retinal Model Implementation

The main motif of this study is to examine the effect of the wide RF component of the retinal interneurons by adding and removing the component in the biophysical retinal models and assessing how this changes the spatial induction patterns in the model responses. The patterns of the model responses are compared to those of the psychophysical data.

To make a fair comparison between the model responses and human perception data, we presented the model retinas with the configurations of stimuli identical to those used for psychophysical experiments, and designed the simulation procedures as intuitive and straightforward parallels to the behavioral experiments. The models by Wilson [36] and by van Hateren [37,38] were chosen to meet these needs among available retinal models [40–42], since both of the models take in realistic input (2-D images in projected retinal sizes and troland intensities) as stimulus.

We compared the results across two different retinal models, each with distinct algorithmic design, rather than testing on a single model in order to ensure the computational robustness of our claim. This way, we intended to identify coherent mechanical features with which both of the models predict the brightness induction data patterns regardless of the algorithmic details of each model.

The most critical difference between the two models for the purpose of the current study is that Wilson’s model [36] did not incorporate the wide interneuron RF component, whereas van Hateren’s model [37,38] did, and neurophysiologically validated it. Thus, we added the wide RF structure to Wilson’s model and compared the model behavior with that of the original model in its native algorithm (without wide RF) as well as with that of van Hateren’s model (with wide RF). We also removed the wide RF component in van Hateren’s model and compared the results with that of the original model (with wide RF).

Also, Wilson’s model [36] contains a comprehensive retinal structure embodying all the main layers and parallel processing pathways but this model computes the cell responses in a mathematically abridged way (less precise quantitative fit to neurophysiological data). van Hateren’s model [37,38] embodies only photoreceptor and horizontal cell layers but consists of complicated algorithmic processes of the internal dynamics of each cell class such that the model cell responses quantitatively match a large collection of neurophysiological data.

The implementation and modification of the models in the current study aimed to best assure that we do not adapt the retinal models merely for the purpose of obtaining a better data fit. Since Wilson [36] and van Hateren [37,38] rigorously validated each of their models for neurophysiological feasibility, we implemented the models in their native algorithmic structures with the native parameters, except for the sizes of the interneuron RF that were the key modifications for our study. For van Hateren’s model, since this is a photoreceptor-horizontal cell partial circuitry model, we minimally augmented the model with the spatial processing structure of the parasol pathway.

In the following subsections, we concisely introduce the original models by Wilson and by van Hateren and describe our main modifications and other implementation details. We refer to the original studies for most of the algorithmic details of the models.

The generic simulation setup is provided in full detail in **S1 Appendix**.

### The Model by Wilson

#### Overall model architecture

**Fig 3:**
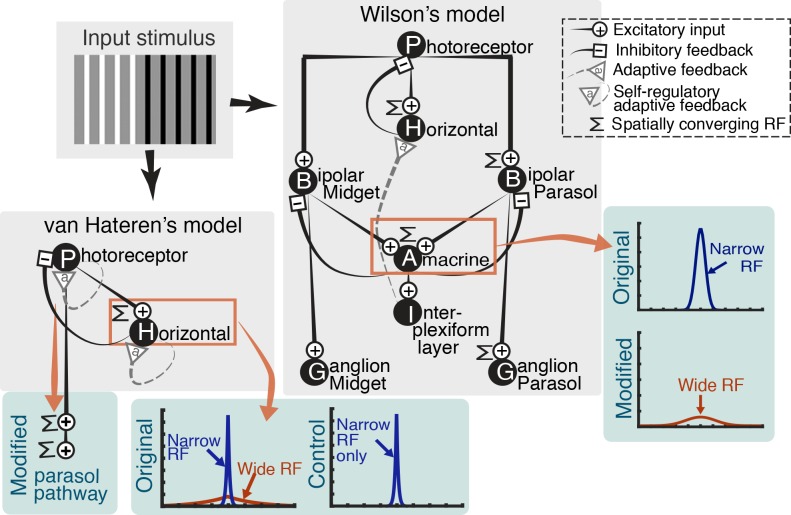
Diagrams of Wilson [36] and van Hateren [37,38] model structures and the modifications made to the models in the current study. Grey box shows the original versions of the models. Modifications made in the current study are shown in green boxes led by the red arrows. See text for details.

The retinal model by Wilson [36] demonstrates the functional architecture of the retinal circuitry (Fig 3, Wilson’s model). The model embodies a full set of basic anatomical layers (photoreceptors, horizontal cells, parasol and midget ON and OFF bipolar cells, ON and OFF amacrine cells, interplexiform layer cells, and parasol and midget ON and OFF ganglion cells). Photoreceptors and horizontal cells are paired to form a local feedback circuit and, similarly, parasol/midget ON (OFF) bipolar and ON (OFF) amacrine cells are paired. Interplexiform layer cells receive inputs from amacrine cells and send inhibitory signals back to horizontal cells thereby accomplishing a long-range feedback that adaptively adjusts the gain and speed of horizontal cell responses. These local and long-range interneuron feedbacks regulate the feedfoward signals transmitted along the feedforward hierarchy and accomplish lateral inhibition, contrast gain control, and light adaptation. The center-surround form of the ganglion cell RF arises as a result.

#### Spatial structure of the model cells

In Wilson’s original model [36], the spatial extents of the inhibitory surround, that is, the inhibitory interneuron RF sizes, are within the narrow- to small-field range. The RF is defined as a 2-dimensional Gaussian unit point-spread function, $e^{-r^{2}/\sigma^{2}}$, where *r* is the distance from the RF center and *σ* is the spatial constant in visual angle (*σ* is 0.08° for horizontal cells and 0.15° for amacrine cells).

In the feedforward hierarchy, the parasol pathway signals spatially converge at the bipolar cell (averaging three neighboring photoreceptor inputs) and at the ganglion cell (a Gaussian summation with spatial constant of 0.033°) synapses. In the midget pathway, signals are not spatially converged (one-to-one synaptic correspondence along the feedforward hierarchy).

#### Pertinent modifications for the current study

**Fig 4:**
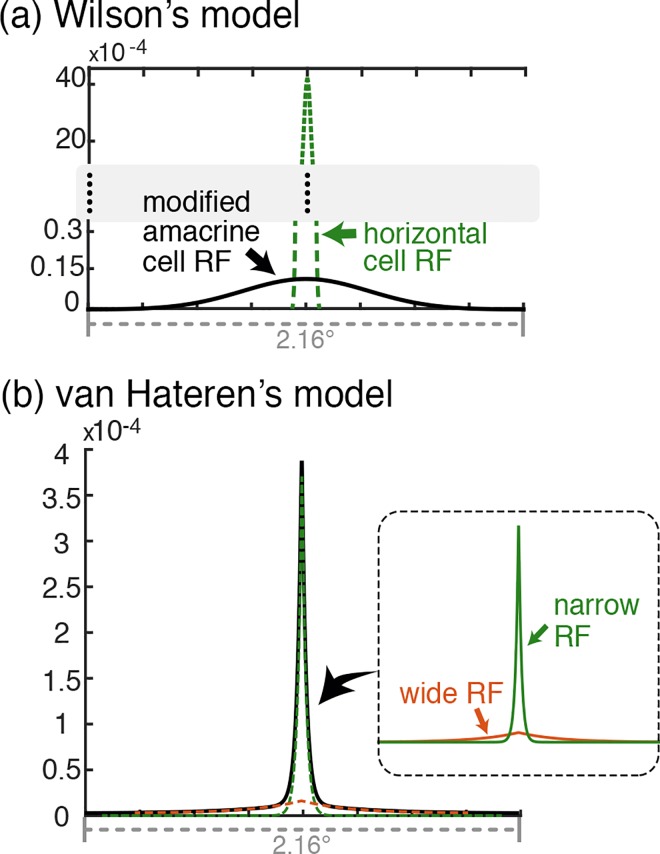
Illustration of the wide RF profiles in the retinal models. (a) Wilson’s model. The amacrine cell wide RF profile (solid black line) is a modification introduced in this study. The dotted green line shows the horizontal cell filter profile (narrow RF) in the same scale for comparison. (b) The horizontal cell narrow + wide RF in van Hateren’s model (the RF structure is directly taken from [30]). The solid black line illustrates the actual dual-component RF profile and the dotted lines in the plot and the solid lines of the corresponding colors in the inset separately shows the narrow (green) and the wide (red) components for comparison.

In the current study, we incorporated the wide RF component of retinal interneurons into Wilson’s model [36] by increasing the size of the amacrine cell RF. The wide RF size was set to roughly correspond to the size of the wide horizontal cell RF in van Hateren's model (spatial constant of 1.47°) for direct comparison. The RF profile used in the simulation is plotted in Fig 4A (solid black line) together with the horizontal cell RF (dashed green line) in the same scale for comparison.

In the primate retina, the types of different amacrine cells are abundant with many of them anatomically classified as wide-field cells (dendritic field diameter > 500 μm). However, the functional roles and precise synaptic connectivity structures of varying types of amacrine cells are subject to future studies and how far and which type of these cells could shape the retinal spatial processing as we demonstrate through the model are open questions [31].

#### Minor implementation details

In the original study by Wilson [36], all the simulations were performed in 1-dimensional space merely to reduce computation time while the model algorithms were readily extendable to 2-dimensional simulations. We did extend Wilson's model and performed all the simulation in 2-dimension. Note that this extension slightly changes Equation (4) in the original article [36] (a bipolar cell averages three neighboring photoreceptors’ inputs in the 1-dimensional simulations, but this changes to averaging 3x3 neighboring photoreceptors in the 2-dimensional simulation).

Otherwise, our model implementation adhered to the original study [36].

### The Model by Van Hateren

#### Overall model architecture

van Hateren [37,38] developed a state-of-the-art partial retinal circuitry model that implements a cascade of phototransduction processes in photoreceptors (see also [43]) and the inhibitory feedback from the horizontal cells to photoreceptors, which gives rise to the center-surround RF in photoreceptor responses.

Note that the initial version of the model [37] is thoroughly tested regarding its performance to emulate realistic temporal dynamics of horizontal cell responses, and van Hateren later improved the model by adding spatial processing RF structures as well as the light-adaptive properties of the horizontal cells (refer to Fig 1C in [38]). The two versions of the model slightly differ in terms of the mathematical details and here we implemented the latter version [38].

#### Spatial structure of the model cells

The horizontal cell RF in van Hateren’s model is directly taken from neurophysiological studies [29,30,44], showing that the dual-component RF profile is well represented as a weighted sum of two exponential unit point-spread functions (*e*^−*r*/*λ*^; *r* is the distance from the RF center; *λ* is a spatial constant in μm; for more details about the RF structure, see the section ‘A two-component spatial receptive field’ in [38]), one exponential function with the small spatial constant (narrow RF component) and the other with the large spatial constant (wide RF component) as illustrated in Fig 4B. The narrow RF component results from the direct dendritic connection from photoreceptors to horizontal cells, while the wide RF component results from electric coupling among adjoining horizontal cells. In the original study of van Hateren, the suggested generic parameter values for these spatial constants were 20 μm in radius for the narrow RF component and 300 μm for the wide RF component. We used these generic values in the current study.

#### Pertinent modifications for the current study

Since van Hateren only modeled a partial retinal circuitry, an additional computational process was needed to introduce the spatial convergence of the parasol pathway where luminance processing is largely accomplished [45–47]. As a minimal treatment for this purpose, we passed the model photoreceptor responses through two stages of spatial convolution with each stage representing the RF of diffuse bipolar cells and of parasol ganglion cells. We defined the corresponding RF structures (i.e. spatial filter shapes) to be identical to those used in Wilson’s model (a 3 x 3 grid averaging filter for a bipolar cell and a Gaussian filter with the standard deviation of 0.033° for a ganglion cell).

To confirm the necessity of the wide RF component of horizontal cells in reproducing the perceptual phenomena of our interest, we additionally performed control simulations, in which we removed the wide RF from the model. We also conducted the same simulations when the narrow RF component was removed to isolate the wide RF effect. In these control simulations, we simply generated the filters with a single exponential function.

#### Minor implementation details

While the original study used autoregressive-moving-average filtering in space coordinates, we simplified the computational process by performing the filtering in the frequency domain (see **S1 Appendix**).

For computational convenience, we omitted one minor enhancement that was introduced in the later version of the model, the adaptive temporal filtering feature (Fig 1C in [38]) implemented for a slight quantitative improvement of model responses for high temporal-frequency stimulations (80 Hz) of a large stimulus with a homogeneous surface (10°). Since such temporal precision at high temporal frequencies is irrelevant in the context of the current study, however, we bypassed this feature.

In all other respects the implementation is exactly as in the original, including the parameter values.

## Results: Comparison of Outputs of Retinal Models with Psychophysical Data

We demonstrate the effect of the wide interneuron RF component by simulating psychophysical experiments on simultaneous brightness induction in the two biophysical retinal models described above. We targeted three psychophysical experiments: two classic experiments on brightness assimilation-to-contrast as a function of increasing surrounding surface size by Helson [11] and by Reid and Shapley [12] and a long-range brightness induction experiment by Rudd and Zemach [39] that systematically measured the magnitude of brightness induction as a function of spatial extent of the surrounding surface. Before presenting the details of the model simulation results, we briefly introduce these psychophysical studies here.

**Fig 5:**
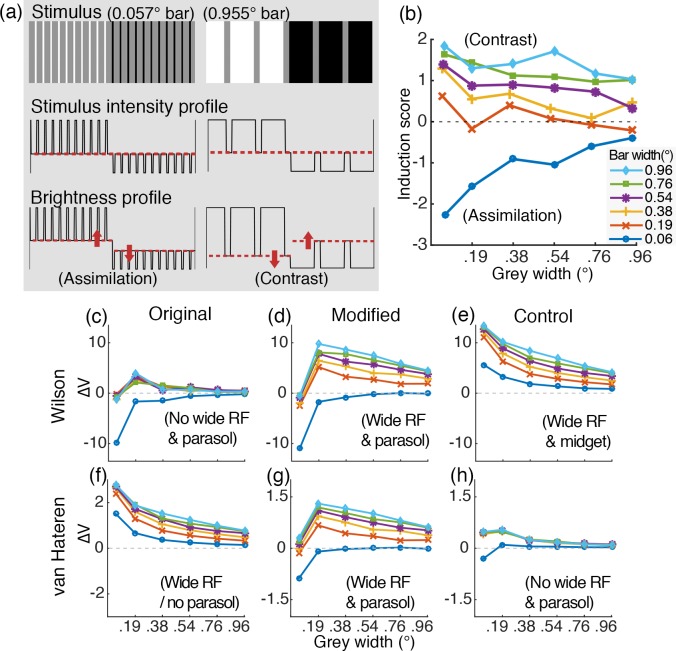
Behavioral and simulation results of Helson’s experiment [11]. (a) Examples of stimuli with narrow bars (left) and wide bars (right). The comparison of stimulus intensity profile with brightness profile schematically illustrates how brightness assimilation and contrast are induced, given narrow bars and wide bars, respectively. (b) The behavioral data on the induction direction and magnitude as a function of grey width for different sizes of bars (replotted from Fig 2 in [11]). A negative / positive induction score indicates assimilation / contrast. (c-h) The retinal model simulation results. (c-e) for Wilson’s model [36] and (f-g) for van Hateren’s model [37,38], with the original algorithms (c, f), modified versions (d, g) and control versions (e, h). All are plotted comparable to (b) except that y-axis is ∆V (mean responses to the grey area among white bars—mean response to the grey area among black bars). Wide RF + parasol spatial processing structures are necessary to predict the bright assimilation-to-contrast induction with increasing bar widths comparable to (b).

Helson [11] investigated brightness induction by varying the width of bars (refer to Fig 5A). Using a stimulus comprised of a set of white bars (left half of stimulus) and a set of black bars (right half of stimulus) that were drawn on a homogeneous grey surface, he measured the direction (assimilation vs. contrast) and magnitude of the induction when the widths of the white and black bars were varied. The results showed that the grey area among white bars was perceived to be lighter than that among the black bars (brightness assimilation) when the bars were narrow. However, when the white and the black bars were sufficiently wide, the brightness of the grey area among the white bars appeared darker compared to that among black bars (brightness contrast) and stronger contrast induction was observed with increasing bar width. These results suggest that increasing the spatial scale of the white and the black bars gradually changed the direction of induction from assimilation to contrast.

**Fig 6:**
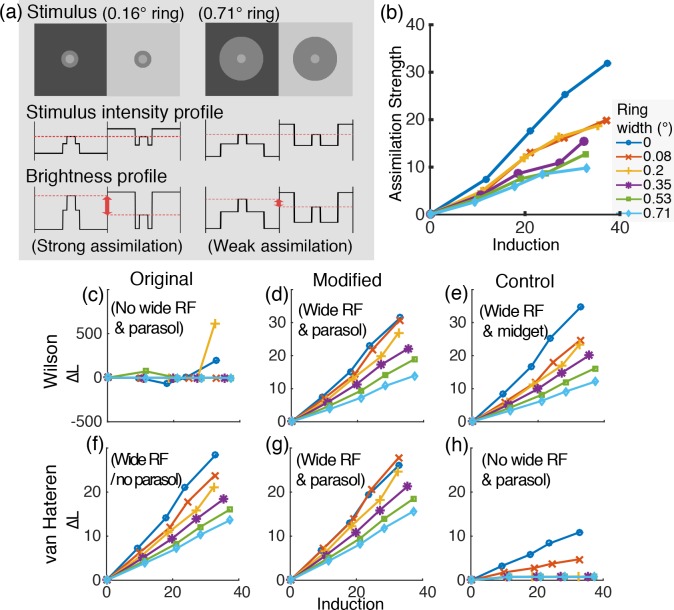
Behavioral and simulation results of Reid and Shapley’s experiment [12]. (a) Examples of stimuli with a narrow ring (left) and a wide ring (right). The comparison of stimulus intensity profile with brightness profile schematically illustrates how a narrower ring induces stronger assimilation compared to a wider ring. (b) The behavioral data on the assimilation strength as a function of difference of the background luminance (labeled ‘induction’ following the terminology in [12]) by different sizes of rings (replotted from Fig 3 in [12]). Larger values indicate stronger assimilation. (c-h) The retinal model simulation results. (c-e) for Wilson’s model and (f-h) for van Hateren’s model, with the original algorithms (c, f), the modified versions (d, g) and the control versions (e, h). All are plotted comparable to (b) except that y-axis is ∆L (the luminance of the disk in the lighter background minus the baseline condition result). The wide RF component is necessary to predict the stronger assimilation magnitude by the narrower rings as in the behavioral data illustrated in (b).

Reid and Shapley [12] reported another evidence of the surrounding-size-dependent brightness assimilation-to-contrast by different widths of rings surrounding a disk (refer to Fig 6A), which verified their dual-mechanism hypothesis on assimilation-to-contrast effect (see Introduction). They presented two identical disk-and-ring stimuli, both with the same physical amount of luminance contrast between the disk and the ring, one on a dark and the other on a light background field, and compared the brightness of the disks in the two stimuli. This method allowed to exclusively quantify the magnitude of assimilation (the influence of the background luminance onto the disk brightness through long-range interaction) induced by different widths of rings while the amount of brightness contrast (brightness induction by the ring to the disk by local contrast) was fixed. The results showed that the brightness of the disk in the dark background appeared lighter than the one in the light background (assimilation) but the magnitude of this assimilation (difference of the brightness between the two disks) gradually decreased as a function of increasing ring width, supporting that increasing the size of the surrounding surface decreases assimilation and increases contrast.

Rudd and Zemach [39] also investigated the effect of the ring width using the same disk-and-ring display and experimental method as Reid and Shapley [12] did, but, rather than investigating assimilation, they examined the magnitude of brightness induction directly produced by different widths of the rings. For this, they measured the brightness of the disk as Reid and Shapley except that they varied the target ring luminance (direct manipulation of the magnitude of local contrast) instead of the background luminance difference (manipulation of the magnitude of the long-range induction effect). The results showed that the magnitude of the induction by the ring onto the disk increased with increasing ring luminance and the rate of the induction magnitude increment was steeper for wider rings. The rate difference appeared over the entire ring width range they tested, which suggests that this effect should be regulated by a long-range interaction extending to several degrees.

### Helson (1963): Assimilation-to-Contrast by Increasing Bar Width

#### Summary of psychophysical experiment

Helson [11] conducted a behavioral study in which sets of black and white bars of varying width (0.06°, 0.19°, 0.38°, 0.54°, 0.76°, 0.96°) were drawn on a homogeneous 3.4° x 5.33° rectangular grey field of 36% reflectance and the induction by the bars onto the grey area was measured. The grey area width (i.e. distance between two neighboring bars) was also manipulated (0.06°, 0.19°, 0.38°, 0.54°, 0.76°, 0.96°; note that all the different unit notations in the original behavioral studies are converted into visual angle degree for stimulus size and *cd/m*^*2*^ for stimulus intensity throughout the text for coherence). The induction effect was measured by rating how much darker (lighter) the grey appears among white bars compared to that among black bars, with higher (lower) score indicating more contrast (assimilation). Fig 5B re-plots the phychophysical data reported in [11] (Fig 2; we rescaled the original plot so that the negative values on the y-axis indicate assimilation and the positive values contrast), which shows this induction score as a function of grey area width for different bar widths. Demonstrating the bar width effect, a wider bar induced stronger contrast but the strength of the contrast was reduced and ultimately reversed to assimilation as the bar width decreased.

#### Simulation method and procedure

The stimuli for the model simulations were generated in identical retinal-projection sizes as in Helson's study [11]. Since the absolute luminance values for black and white bars and grey area were not provided in the original study and only the reflectance of the grey area was reported, we set the input stimuli intensity by first choosing the mean luminance intensity, which is assumed to be the mean display luminance (i.e. adapting light intensity; we arbitrarily set this value to 30 *cd/m*^*2*^), and assuming this value to be the mean luminance level that corresponds to the 50% reflectance grey point (other mean luminance values were tested and confirmed not to affect the results). Then values for the black and the white bars were set to yield Michelson contrast of 90% around this mean luminance level such that the black bars have reflectance of 5% (3 *cd/m*^*2*^) and white bars 95% (57 *cd/m*^*2*^) with the homogeneous-illumination assumption. The luminance of the grey region between the black and white bars was set to 36% (22 *cd/m*^*2*^) as used by Helson.

The 36 stimulus conditions (6 bar width x 6 grey width) were simulated and the mean activities of the model cells whose RF locations correspond to the grey stimulus area were computed for each condition. Brightness induction was scored by subtracting the mean voltage of the cells responding to the grey area among white bars from the mean at grey among black bars (∆V; positive / negative values indicate contrast / assimilation). Since ∆V is the relative mean voltage difference index, the relative relation of ∆V among different conditions, rather than the absolute value of ∆V in each condition should be considered. Also, note that the absolute value of the ∆V should not be directly compared to the y-axis of the psychophyscial data that indicates the rank score value (Fig 5B).

For Wilson's model, we analyzed parasol ganglion cell responses as a standard, for these cells are known to code luminance information [45–47]. On the other hand, since van Hateren's model only contains photoreceptors and horizontal cells, we first analyzed the photoreceptor responses as the original model output. Then we imitated the parasol pathway spatial processing structure by passing photoreceptor responses through a two-stage spatial low-pass filter.

#### Simulation result

The simulation results produced by the two models using their original algorithms were different, and neither of them matched Helson's data. The parasol ganglion cell response pattern from Wilson's model did not show any distinguishable bar size effect on the magnitude of brightness induction (for the widths of 0.19°-0.96°), except for the narrowest bar (0.06°) that induced assimilation (Fig 5C). On the other hand, the photoreceptor responses of van Hateren's model did show a distinguishable size effect among different bar widths, but the model did not produce assimilation for the narrowest bar (Fig 5F).

On the other hand, with the proper modifications to the models, the responses of both of the models matched the psychophysical data. When Wilson's model was modified to include a wide RF for the amacrine cells, then it produced a bar width effect comparable to Helson's data (Fig 5D), showing gradual increase of the magnitude of the contrast induction by increasing the bar width from 0.19° to 0.96°. Likewise, when we added the two-stage spatial convergence of the parasol pathway to van Hateren’s model, the parasol cell responses of the model showed the assimilation for the narrowest bar (0.06°) (Fig 5G). These results suggest that the wide RF component of the interneurons is essential to produce the systematic effect of the bar width on brightness assimilation-to-contrast phenomena. We verified this point again by performing the simulations for the same experiment when the wide RF component in van Hateren's model was removed (parasol cell responses; Fig 5H), which eliminated the systematic effect of the bar width on the magnitude of the induction.

On the other hand, assimilation by the narrowest bar (0.06°) required the parasol pathway spatial convergence. The midget ganglion cell responses in Wilson’s model (Fig 5E) did not show assimilation for the narrowest bar (0.06°) similarly to the photoreceptors of van Hateren’s model (Fig 5F).

The simulation results of the modified retinal models (Fig 5D and 5G) overall showed a good match with the behavioral data (Fig 5B), except for the condition of the narrowest width of the grey area (i.e. the left-most data point for all the bar width conditions in Fig 5B, 5D and 5G). The mismatch at this data point is probably resolved if the combined effect of the parasol and the midget pathway processing on perception is considered. Although encoding of luminance information in the visual system depends mostly on the parasol pathway processing [45–47], the parasol pathway spatial summation is too crude for a higher-end spatial-frequency bandwidth of information and such information needs to be represented in the midget pathway [21,23,45]. However, how the midget and the parasol pathway signals are combined in the visual system is still an open problem. To avoid making any assumptions without a conclusive neurophysiological justification, we separately provide both the parasol and the midget pathway results.

### Reid and Shapley (1988): Assimilation-to-Contrast by Increasing Ring width

#### Summary of psychophysical experiment

This experiment measured the magnitude of assimilation (the influence of background onto the disk brightness through long-range interaction) by varying ring widths (0°, 0.08°, 0.2°, 0.35°, 0.53°, or 0.71°) while the amount of brightness contrast (brightness induction by the ring to the disk by local contrast) was constant [12]. Reid and Shapley presented two disk-and-ring stimuli of identical luminance composition (the disk luminance was 78 *cd/m*^*2*^ and the ring luminance and the mean display luminance were 70 *cd/m*^*2*^), one on a dark and the other on a light background field. Observers adjusted the luminance of the disk in the lighter background (D_B_light_) to match the disk brightness in the darker background (D_B_dark_). The difference of background luminance for each stimuli pair was systematically manipulated ({70, 70}, {65, 74}, {61, 78}, {57, 82}, and {53, 86} *cd/m*^*2*^ with the {70, 70} set as the baseline condition in which assimilation strength = 0, i.e. the same background luminance for both background fields). The assimilation strength was indexed as the adjusted luminance of D_B_light_ subtracted by that of the baseline condition. Fig 6B presents this assimilation strength index, replotted and re-labeled from Fig 3 in [12] (the average data of four subjects), which demonstrates that the luminance of the matched disk (D_B_light_) increased as a function of increasing luminance difference of the background fields for each ring width. For wider rings, however, the background effect was smaller (lower slope) than the narrower rings; thus, the narrower the ring is, the stronger the assimilation.

#### Simulation method and procedure

The input stimuli to the model simulations were entirely identical to those in the behavioral study.

For the analysis of the model outputs, the mean voltage of model cells responding to the disk was set as the indicator of the disk brightness and we determined the luminance of D_B_light_ such that the mean voltage of the model cell responses to D_B_light_ equals to the mean voltage responses to D_B_dark_.

The simulation procedure needed to be slightly modified with respect to the procedure of the behavioral experiment to be suitable for a simulation environment, in which observers were presented with the target and the match stimuli sets simultaneously and they continuously compared the appearance of the match disk (D_B_light_) and the target disk (D_B_dark_). For the model simulations, this adjustment process was imitated by performing simulations for a large match disk luminance range for the match stimulus set (in log luminance range from -1 to 1 around the target disk luminance with a unit step size of 0.2 log units; 11 steps in total) per each of the 30 conditions (6 ring widths x 5 background luminance pairs). We then computed the mean of the model cell activities responding to the match disks for each of the 11 luminance-steps, and fitted these results with a 5^th^ order polynomial function (i.e. the mean model cell response as a function of match disk luminance). The mean cell response was computed for the target disk of the corresponding condition as well, and we searched for the luminance of the match disk that yielded the same mean response as did the target disk.

As in the psychophysical study, the baseline luminance was subtracted from the determined D_B_light_ luminance, which is equivalence of the assimilation strength index (∆L; larger values indicate stronger assimilation). Thus, what the y-axis indicates is identical between the psychophysical data (Fig 6B) and the model simulation results (Fig 6C–6H) and the absolute values can be compared in this case.

#### Simulation result

Comparison of the cell responses between the two original models without any modifications (parasol ganglion cell responses for Wilson's model and photoreceptor responses for van Hateren's model) showed that van Hateren's model readily reproduced the varying magnitude of assimilation (∆L) by the different ring widths, explaining the ring size effect on the brightness of the disk as a function of background luminance difference (Fig 6F), while Wilson's model did not (Fig 6C). Comparably, however, modifying Wilson's model to include the wide amacrine cell RF produced the ring size effect matching the psychophysical data (Fig 6E).

Eliminating the wide RF component in van Hateren's model removed the systematic ring size effect on assimilation (Fig 6H). In this case, assimilation was induced only for the two narrowest rings. This result suggests that the narrow interneuron RF alone cannot explain the systematic ring size effect over the entire ring width range, which is consistent with an earlier computational study by Heinemann and Chase [5] (see Discussion).

The synaptic convergence in the parasol pathway was not critical in predicting the overall ring size dependency of the assimilation magnitude in the data pattern in this case (Fig 6D and 6E and Fig 6F and 6G).

Our results demonstrate that the wide RFs of retinal interneurons indeed account for assimilation in Reid and Shapley's data. Moreover, van Hateren's model results imply that this effect could in fact occur at the very first stage of visual processing at the photoreceptor level.

### Control Simulation: Isolated Effect of Wide RF

In the previous sections, we compared the model stimulation results when a wide RF was included or not in the retinal models and showed that the wide RF component of the interneurons was necessary to predict the brightness assimilation-to-contrast induction patterns as functions of increasing surround size in the studies by Helson [11] and Reid and Shapely [12].

In this section, we show that the wide RF component is sufficient to predict most of the surrounding size effect with van Hateren’s model [37,38] by simulating the same experiments when the narrow RF component of the horizontal cells was eliminated. We did not perform this simulation with Wilson’s model since the horizontal cells in Wilson’s model have a complex functional structure (i.e. the long-range feedback from the interplexiform layer cells that governs the light adaptive properties of the entire retinal circuitry [36]) such that it was not possible to remove the horizontal cells or to drastically increase their RF size without an alternative mathematical solution to stabilize the system.

**Fig 7:**
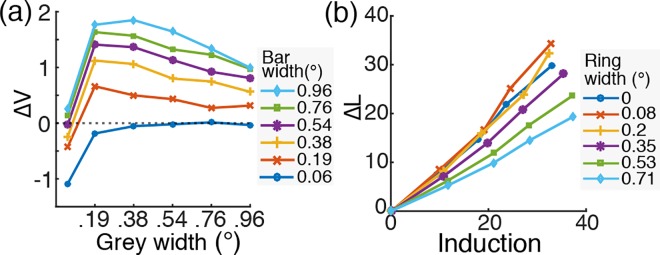
Simulation results of van Hateren’s model with only the wide RF component of horizontal cells. (a) The result for Helson’s experiment [11]. (b) The result for Reid and Shapley’s experiment [12]. See and compare with Figs 5G and 6G, respectively.

The simulation methods and procedures were identical to previous simulations except for the removal of the narrow RF component from the horizontal cell RF. The results are plotted in Fig 7. The patterns of the simulation results are comparable to the results in the previous sections (Fig 5G for Helson and Fig 6G for Reid and Shapley), showing clear systematic effects of the different bar widths on induction direction (i.e. assimilation or contrast) and magnitude (Fig 7A) as well as the varying effect of the ring size on the assimilation strength (Fig 7B). This suggests that the wide RF was mostly responsible for producing the cell responses matching the assimilation-to-contrast behavioral patterns.

### Rudd and Zemach (2004): Spatial Extent of Wide RF Effect

#### Summary of psychophysical experiment

Rudd and Zemach performed an experiment very similar to the experiment by Reid and Shapley, but with varying target ring luminance instead of the luminance difference of the background fields. The target ring luminance varied in six steps from 2.56 to 6.31 *cd/m*^*2*^. Nine different ring widths were tested (0.06°, 0.18°, 0.35°, 0.70°, 1.06°, 1.41°, 1.77°, 2.13°, and 2.48°). The target disk luminance and the match ring luminance were set to 1.02 *cd/m*^*2*^ and 3.94 *cd/m*^*2*^, respectively, and the observers adjusted the luminance of the match disk to appear in the same brightness as the target disk as in Reid and Shapley’s experiment. The stimulus sets were presented on a 0.1 *cd/m*^*2*^ background field.

Rudd and Zemach [39] computed the slope of the log match disk luminance (adjusted) against log luminance of the target ring (varying; independent variable). Note, if the target ring renders the target disk to appear darker by inducing local contrast, the match disk luminance would decrease with increasing target ring luminance (the slope is negative). Also, if a narrower ring generates a weaker local contrast effect, the slope of the narrow ring would be shallower (the absolute value of the slope is smaller) than a wider ring. Finally, if the spatial extent of the ring size effect has an upper limit beyond which the ring width no longer alters the induction magnitude, the slope decrement with increasing ring width would reach a plateau after this upper limit.

**Fig 8:**
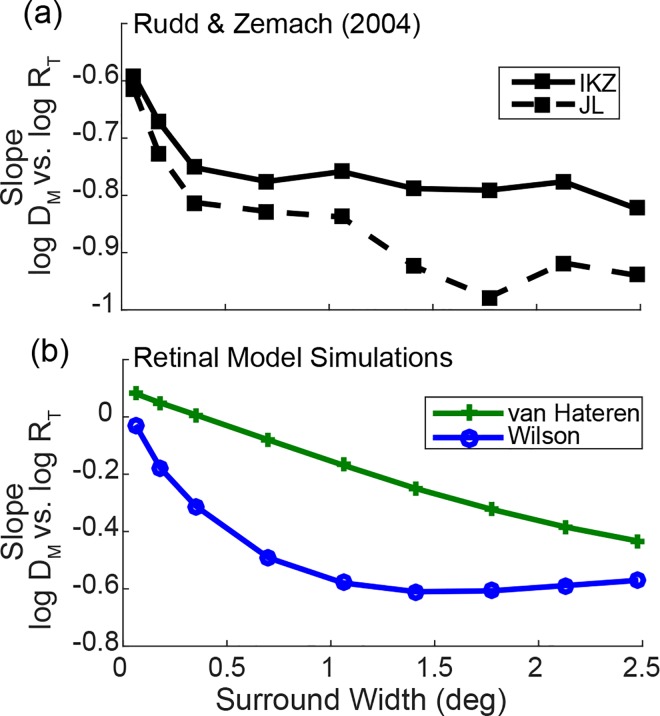
Behavioral and simulation results of Rudd and Zemach’s experiment [39]. (a) The behavioral data on the slope of the log match disk luminance vs. log target ring luminance for two subjects, IKZ and JL (see text for details). (b) The simulation results for the same experiment. The green line with ‘+’ markers represent the result of van Hateren’s model and the blue line with ‘o’ markers represent the result of Wilson’s model.

Fig 8A re-plots Fig 3 from [39] that shows the slopes of log match disk luminance against log target ring luminance as a function of the ring size for the two observers tested. The results indicated that the magnitude of the ring luminance effect increased with increasing ring width, although the plateau of the slope change differed between the two observers.

#### Simulation method and procedure

We simulated this same experiment in our modified versions of Wilson’s and van Hateren’s models. All the stimulus aspects were identical to the psychophysical experiment except that the luminance step size for the target ring luminance was linear in the 8-bit intensity (i.e. RGB channel values of the image) scale in the original study, and we set them linearly in log step for the simulation. The simulation procedure was identical to those for the simulation of Reid and Shapley’s experiment in that we determined the luminance of the match disk such that the mean model cell responses coinciding at the target disk location of the visual field and the mean model cell responses at the match disk location are equal. We then performed the same slope analysis on the obtained match disk luminance that Rudd and Zemach applied to their psychophysical data. As in the simulation of Reid and Shapley’s experiment, the absolute slope values (y-axis) of the psychophysical data plot (Fig 8A) and the model simulation results (Fig 8B) are directly comparable in this case.

#### Simulation result

Fig 8B plots the results of the same slope analysis for the retinal cell output in our simulations (parasol ganglion cells; with the wide interneuron RF in both of the retinal models). The results show a distinguishable effect of the ring width up to or beyond ~1° comparable to the behavioral data, although the two models produced different results as to the rate of slope decrement for increasing ring width and also to the plateau point of the slope change (similarly to the behavioral results). The detailed shape difference of the results between the models and the quantitative deviation of the model results from the behavioral results might be due to the specific algorithmic architecture of each model (e.g. RF or gain control design) or to the omission of some luminance-dependent gain control processes that occur beyond the architectures of the models (e.g. the amacrine cell circuit for the case of van Hateren’s model, or other retinal or post-retinal mechanisms for both of the models). While understanding these matters may be important in analyzing and improving the retinal model architectures, we leave these discussions for future studies since the current work focuses only on demonstrating the long-range interaction of wide interneuron RF. Nevertheless, the overall results suggest that the wide interneuron RF can produce the long-range spatial interaction that extends ~1° or beyond.

## Discussion

We simulated brightness induction experiments in two different biophysical retinal models and showed that the effect of the size of the surrounding surface on brightness assimilation-to-contrast phenomena observed by Helson [11] and Reid and Shapley [12] is reproduced when the wide RF component of the interneurons is incorporated in the models. We further showed that most of the surrounding size effect could be explained exclusively by the wide RF component. Moreover, the spatial extent to which the wide RF affects the induction phenomena was comparable to the perceptual data by Rudd and Zemach [39].

The architectures of the two retinal models are based on neurophysiological data both anatomically and functionally, and all the spatial processing structures in the original models including the wide RF component of horizontal cells in van Hateren's model were designed to essentially reproduce the response properties of the real retinal cells.

Even though neither of these retinal models is a complete replica of the true retinal circuitry, the current results support our main argument. If there exists a wide RF component for the retinal interneurons (regardless of the type, horizontal cells or amacrine cells) that send inhibitory signals to the retinal feedforward cells, the long-range effect observed in the perceptual phenomena of spatial induction can be explained at the retinal level. There are many neurophysiological reports on the existence of the wide RF component. Therefore, we conclude that the long-range effect is very likely to occur at the retinal level. Thus, our results provide compelling evidence that the retina contains sufficient spatial processing structures to produce the surrounding-size dependency in the simultaneous brightness induction phenomena.

At the same time, it should be noted that our claim on the retinal origin of the simultaneous brightness induction is limited to the surrounding size dependency. Especially, assimilation is further shown to involve post-retinal mechanisms including orientation/depth processing and attention [13,48–58]. Indeed, the output signals from the retina are subject to more complex information processing in the cortex and brightness computation must result from this whole range of processing. Meanwhile, our results show that the retina is a significant starting point of the spatial induction including assimilation.

The results of the current study raise questions on the traditional lateral inhibition theories that are based on the classic RF. The classic RF assumption omits the wide RF component of the retinal interneurons, which seems to be the reason that the neural mechanism underlying surrounding size dependency of brightness induction has been left largely unexplained. We propose that incorporating the wide RF component to the classic RF is a simple and effective update to the traditional view on lateral inhibition.

In fact, the classic RF traditionally assumed in the context of lateral inhibition is in discord with the later neurophysiological discoveries on the ganglion cell’s response characteristics, which exhibit an extra-classical suppressive RF surround component [21,32–35]. The reason this extra-classic RF component was not considered for the ‘classic’ ganglion cell RF might be simply because the early stage studies on the retinal ganglion cells (to which the classic RF is based on) focused on the robust, thus, more detectable RF components (the center and the narrow surround), bypassing any weak peripheral effect (the extra-classic surround). As of yet, little has been discussed about the wide RF component of the interneurons in the literature beyond its existence, and evidence has been lacking to shed light on how the extra-classic RF is formed or what is the functional role of the wide RF component or the extra-classic RF surround (but see [31]). Our results suggest that the wide interneuron RF can affect ganglion cell response patterns and function to operate the long-range cellular communication that is comparable to the ‘global response mean computation.’

We may deduce a mechanical interpretation on the functionality of the wide RF component by comparing our results to the earlier computational model by Heinemann and Chase [5] on simultaneous brightness induction. Heinemann and Chase’s model was comprised of the following three computational stages: The lateral inhibition stage (the input stimulus is spatially filtered through a DOG function, i.e. the classic RF), the response nonlinearity stage (the output of the lateral inhibition stage is thresholded and logarithmically scaled), and the global adaptation stage (the output of the response nonlinearity stage is subtracted by its global mean). According to their model, the ring width effect in Reid and Shapley's experiment resulted mostly from the global adaptation stage (since decreasing the ring width reduces the contribution of the ring luminance to the global mean), whereas there was no significant influence of the ring width in the outputs of the lateral inhibition or the response nonlinearity stages, except for the smallest ring width (0.08°).

These results are comparable to our simulation results in that the models with only the wide RF component, even without the narrow RF component, could produce most of the ring width effect (Fig 7B), while the narrow RF only generated a distinctive ring width effect for the narrowest widths (Fig 6H). Thus, the wide RF component yields the functional consequence equivalent to the global mean computation in Heinemann and Chase’s model.

The current study in fact provides the biological ground to the non-biological assumption made on the global mean computation in Heinemann and Chase’s model [5]. Heinemann and Chase limited the area of the global mean computation to be within 0.5°-1.5° in visual angle based on the psychophysical evidence on the distance of a remote stimulus influencing perception of a foveal stimulus. We likewise found that the wide RF component of the interneurons could generate the spatial effect up to or beyond 1.5° (Fig 8). This evidence in general suggests that the long-range effect of spatial induction could result from the wide RF of the retinal interneurons.

The results of the current study contest the dual-mechanism hypothesis proposed by Reid and Shapley [12]. Their work, in line with other studies [5,25,59,60], postulated that lateral inhibition and long-range surface interaction are two separate mechanisms modulating spatial induction that occur in different stages of the visual processing. However, our results suggest that these seemingly discrete processes can be accomplished by a wide and narrow dual-component RF of retinal interneurons and need not involve a neural mechanism other than retinal center-surround processing. In other words, retinal lateral inhibition regulates both local and long-range spatial induction effects.

The long-range cellular interaction in the retina could in theory be mediated by either horizontal cells (as in van Hateren's model) or amacrine cells (as in Wilson's model). On one hand, various types of wide RF amacrine cells are anatomically identified [26–28], and some studies postulate a type of amacrine cell to be the potential source of the formation of the extra-classic surround [33,35]. Manookin et al. [31] recently showed that a type of amacrine cells (“wiry” amacrine cells) integrates signals over wide spatial range, much exceeding the classic ganglion cell RF, and suggested that they could serve for long-range interaction. On the other hand, a horizontal cell’s sensitivity differs between driving stimuli as large as 5° vs. 10° [29,30] (replicated in van Hateren's model [38]), as neurophysiologically and computationally shown to result from its wide RF component. Horizontal cells are also argued to contribute to the ganglion RF surround to a larger degree than amacrine cells [14,61]. However, no discussion has been made so far regarding any potential contribution of the horizontal cells’ wide RF component on the extra-classic ganglion cell RF surround to the best of authors’ knowledge and whether they contribute to long-range retinal communication is an open question.

For these reasons, the current study suggests a substantially disparate claim on the neural origin of the long-range effect recurrently observed in the spatial induction phenomena across various experimental conditions [12,20,24,25,48–54,59,60,62–66]. Rather than it involving the post-retinal processing as proposed in the previous studies [12,13,20,24], it might occur at the very first stage of the visual processing in the photoreceptor-horizontal cell circuit.

In a more global perspective, our results may be linked to the neural mechanism of the Retinex theory on lightness perception [67–69]. Retinex theory proposes a general framework in which the lightness of a certain area (target) in a visual scene is well predicted by the mean average of chain products of luminance ratios along paths that start at arbitrary points and end on the target area [67]. In a version of Retinex theory, Land [69] proposed to use the average of the luminance ratios of the target with multiple surrounding areas, which is a computation conceptually similar to the center-surround processing. This version of Retinex computation incorporated the contribution of remote surfaces on the target lightness by taking into account the luminance ratio averages over the entire scene. In later Retinex implementations [70,71] and related perceptually based algorithms [72], the influence of the remote surface on the target lightness is weighted as a function of the distance between the target and the surface. The current study proposes that this kind of weighted summation process can be accomplished by wide-RF interneuron feedback. Since there are also works suggesting a connection between Retinex and neuroscience [71,73,74], at present we are working at establishing what kind of relationship there is between those models and the ones employed in this study (for more relevant discussions, see also [13,39,64,75–77] for perceptual evidence and [78,79] for neural evidence).

Our finding proposes alternative perspectives on important classic topics in vision science, such as lateral inhibition, long-range spatial induction, assimilation and simultaneous brightness induction. The current study suggests that lateral inhibition theories need an update to consider the effect of the wide RF component of the retinal interneurons, and this update elucidates the neural mechanism underlying the surrounding size dependency in the spatial induction and explains the assimilation-to-contrast phenomena. Here we provide the primary evidence to initiate revising these important vision science topics and more concrete theoretic effort is required to precisely delineate the impact of the retinal processing.

## Supporting Information

S1 Appendix Generic Simulation Methods. The details of the general simulation set ups on retinal projection, luminance unit, data acquisition and temporal spatial filtering methods are illustrated.
(PDF)

S1 Dataset The complete set of data for the model simulation results. (XLSX)

## References

[1] Barlow HB. Possible principles underlying the transformation of sensory messages. Sens Commun. 1961; 217–234.

[2] Barlow HB. Three points about lateral inhibition In: Rosenblith WA, editor. Sensory communication. Cambridge, MA: MIT Press; 1961 pp. 782–786.

[3] Barlow HB. The coding of sensory messages In: Thorpe WH, Zangwill OL, editors. Current problems in animal behavior. Cambridge: Cambridge University Press; 1961 pp. 331–360.

[4] Goldstein EB. Sensation and Perception. 9th ed. Belmont, CA: Wadsworth Cengage Learning; 2013.

[5] Heinemann EG, Chase S. A quantitative model for simultaneous brightness induction. Vision Res. 1995;35: 2007–2020. 7660605 10.1016/0042-6989(94)00281-p

[6] Jameson D. Opponent-colours theory in the light of physiological findings In: Ottoson D, Zeki S, editors. Central and peripheral mechanisms of colour vision. London: MacMillan; 1985 pp. 83–102.

[7] Ratliff F. Mach bands: Quantitative studies on neural networks in the retina. San Francisco: Holden-Day; 1965.

[8] Ratliff F. Contour and contrast. Proc Am Philos Soc. 1971;115: 150–163.

[9] Shapley R, Enroth-Cugell C. Visual adaptation and retinal gain controls. Progress in Retinal Research. 1984 pp. 263–346.

[10] Fiorentini A. Mach Band Phenomena In: Jameson D, Hurvich LM, editors. Visual Psychophysics. Berlin, Heidelberg: Springer; 1972 pp. 188–201.

[11] Helson H. Studies of anomalous contrast and assimilation. J Opt Soc Am. 1963;53: 179–184. 13953661 10.1364/josa.53.000179

[12] Reid RC, Shapley R. Brightness induction by local contrast and the spatial dependence of assimilation. Vision Res. 1988;28: 115–132. 3413989 10.1016/0042-6989(88)90013-2

[13] Rudd ME. How attention and contrast gain control interact to regulate lightness contrast and assimilation: a computational neural model. J Vis. 2010;10: 1–37. 10.1167/10.14.40 21196510

[14] Lee BB, Martin PR, Grünert U. Retinal connectivity and primate vision. Prog Retin Eye Res. 2010;29: 622–639. 10.1016/j.preteyeres.2010.08.004 20826226 PMC3282052

[15] Enroth-Cugell C, Robson JG. The contrast sensitivity of retinal ganglion cells of the cat. J Physiol. 1966;187: 517–552. 16783910 10.1113/jphysiol.1966.sp008107 PMC1395960

[16] Perlman I, Normann RA. Light adaptation and sensitivity controlling mechanisms in vertebrate photoreceptors. Prog Retin Eye Res. 1998;17: 523–563. 9777649 10.1016/s1350-9462(98)00005-6

[17] Thoreson WB, Mangel SC. Lateral interactions in the outer retina. Prog Retin Eye Res. 2012 pp. 407–441. 10.1016/j.preteyeres.2012.04.003 22580106 PMC3401171

[18] Shapley R, Enroth-Cugell C. Visual adaptation and retinal gain controls. Prog Retin Eye Res. 1984;3: 263–346.

[19] Rodieck RW, Stone J. Analysis of receptive fields of cat retinal ganglion cells. J Neurophysiol. 1965;28: 832–849. 5867882 10.1152/jn.1965.28.5.833

[20] De Bonet JS, Zaidi Q. Comparison between spatial interactions in perceived contrast and perceived brightness. Vision Res. 1997;37: 1141–1155. 9196732 10.1016/s0042-6989(96)00250-7

[21] Kaplan E, Benardete EA. The dynamics of primate retinal ganglion cells. Prog Brain Res. 2001;134: 17–34. 11702542 10.1016/s0079-6123(01)34003-7

[22] Benardete EA, Kaplan E. The dynamics of primate M retinal ganglion cells. Vis Neurosci. 1999;16: 355–368. 10367969 10.1017/s0952523899162151

[23] Benardete EA, Kaplan E. Dynamics of primate P retinal ganglion cells: responses to chromatic and achromatic stimuli. J Physiol. 1999;519: 775–790. 10.1111/j.1469-7793.1999.0775n.x 10457090 PMC2269535

[24] Blakeslee B, McCourt ME. Similar mechanisms underlie simultaneous brightness contrast and grating induction. Vision Res. 1997;37: 2849–2869. 9415365 10.1016/s0042-6989(97)00086-2

[25] Shapley R, Reid RC. Contrast and assimilation in the perception of brightness. Proc Natl Acad Sci USA. 1985;82: 5983–5986. 3862112 10.1073/pnas.82.17.5983 PMC390678

[26] Kolb H. Amacrine cells of the mammalian retina: Neurocircuitry and functional roles. Eye. 1997;11: 904–923. 10.1038/eye.1997.230 9537156

[27] Lin B, Masland RH. Populations of wide-field amacrine cells in the mouse retina. J Comp Neurol. 2006;499: 797–809. 10.1002/cne.21126 17048228

[28] MacNeil MA, Masland RH. Extreme diversity among amacrine cells: Implications for function. Neuron. 1998;20: 971–982. 9620701 10.1016/s0896-6273(00)80478-x

[29] Packer OS, Dacey DM. Synergistic center-surround receptive field model of monkey H1 horizontal cells. J Vis. 2005;5: 1038–1054. 10.1167/5.11.9 16441201

[30] Packer OS, Dacey DM. Receptive field structure of H1 horizontal cells in macaque monkey retina. J Vis. 2002;2: 272–292. 10.1167/2.4.1 12678578

[31] Manookin MB, Puller C, Rieke F, Neitz J, Neitz M. Distinctive receptive field and physiological properties of a wide-field amacrine cell in the macaque monkey retina. J Neurophysiol. 2015;114: 1606–1616. 10.1152/jn.00484.2015 26133804 PMC4563022

[32] Alitto HJ, Usrey WM. Surround suppression and temporal processing of visual signals. J Neurophysiol. 2015;113: 2605–2617. 10.1152/jn.00480.2014 25652919 PMC4416570

[33] Passaglia CL, Enroth-Cugell C, Troy JB. Effects of remote stimulation on the mean firing rate of cat retinal ganglion cells. J Neurosci. 2001;21: 5794–5803. 11466451 10.1523/JNEUROSCI.21-15-05794.2001 PMC5130337

[34] Solomon SG, White AJR, Martin PR. Extraclassical receptive field properties of parvocellular, magnocellular, and koniocellular cells in the primate lateral geniculate nucleus. J Neurosci. 2002;22: 338–349. 11756517 10.1523/JNEUROSCI.22-01-00338.2002 PMC6757604

[35] Solomon SG, Lee BB, Sun H. Suppressive surrounds and contrast gain in magnocellular-pathway retinal ganglion cells of macaque. J Neurosci. 2006;26: 8715–8726. 10.1523/JNEUROSCI.0821-06.2006 16928860 PMC2598390

[36] Wilson HR. A neural model of foveal light adaptation and afterimage formation. Vis Neurosci. 1997;14: 403–423. 9194310 10.1017/s0952523800012098

[37] van Hateren JH. A cellular and molecular model of response kinetics and adaptation in primate cones and horizontal cells. J Vis. 2005;5: 331–347. 10.1167/5.4.5 15929656

[38] van Hateren JH. A model of spatiotemporal signal processing by primate cones and horizontal cells. J Vis. 2007;7: 1–19. 10.1167/7.3.3 17461681

[39] Rudd ME, Zemach IK. Quantitative properties of achromatic color induction: An edge integration analysis. Vision Res. 2004;44: 971–981. 10.1016/j.visres.2003.12.004 15031090

[40] Hennig MH, Funke K, Wörgötter F. The influence of different retinal subcircuits on the nonlinearity of ganglion cell behavior. J Neurosci. 2002;22: 8726–8738. 12351748 10.1523/JNEUROSCI.22-19-08726.2002 PMC6757783

[41] Wohrer A, Kornprobst P. Virtual Retina: A biological retina model and simulator, with contrast gain control. J Comput Neurosci. 2009;26: 219–249. 10.1007/s10827-008-0108-4 18670870

[42] Shah S, Levine MD. Visual information processing in primate cone pathways. II. Experiments. IEEE Trans Syst Man Cybern Part B. 1996;26: 275–289. 10.1109/3477.485878 18263029

[43] van Hateren JH, Lamb TD. The photocurrent response of human cones is fast and monophasic. BMC Neurosci. 2006;7: 1–8. 16626487 10.1186/1471-2202-7-34 PMC1464134

[44] Smith VC, Pokorny J, Lee BB, Dacey DM. Primate horizontal cell dynamics: an analysis of sensitivity regulation in the outer retina. J Neurophysiol. 2001;85: 545–558. 11160492 10.1152/jn.2001.85.2.545

[45] Lee BB, Sun H, Valberg A. Segregation of chromatic and luminance signals using a novel grating stimulus. J Physiol. 2011;589: 59–73. 10.1113/jphysiol.2010.188862 20937716 PMC3039260

[46] Lee BB, Pokorny J, Smith VC, Martin PR, Valberg A. Luminance and chromatic modulation sensitivity of macaque ganglion cells and human observers. J Opt Soc Am A. 1990;7: 2223–2236. 2090801 10.1364/josaa.7.002223

[47] Derrington AM, Lennie P. Spatial and temporal contrast sensitivities of neurones in lateral geniculate nucleus of macaque. J Physiol. 1984;357: 219–240. 6512690 10.1113/jphysiol.1984.sp015498 PMC1193256

[48] Anstis S. White’s effect in lightness, color and motion In: Jenkin MRM, Harris LR, editors. Seeing spatial form. New York: Oxford University Press; 2005 pp. 91–100.

[49] Blakeslee B, McCourt ME. A multiscale spatial filtering account of the White effect, simultaneous brightness contrast and grating induction. Vision Res. 1999;39: 4361–4377. 10789430 10.1016/s0042-6989(99)00119-4

[50] Blakeslee B, McCourt ME. A unified theory of brightness contrast and assimilation incorporating oriented multiscale spatial filtering and contrast normalization. Vision Res. 2004;44: 2483–2503. 10.1016/j.visres.2004.05.015 15358084

[51] Blakeslee B, McCourt ME. Brightness induction magnitude declines with increasing distance from the inducing field edge. Vision Res. 2013;78: 39–45. 10.1016/j.visres.2012.12.007 23262229 PMC3556186

[52] Hong SW, Shevell SK. Brightness contrast and assimilation from patterned inducing backgrounds. Vision Res. 2004;44: 35–43. 14599569 10.1016/j.visres.2003.07.010

[53] White M. A new effect of pattern on perceived lightness. Perception. 1979;8: 413–416. 503772 10.1068/p080413

[54] White M. The effect of the nature of the surround on the perceived lightness of grey bars within square-wave test gratings. Perception. 1981;10: 215–230. 7279550 10.1068/p100215

[55] Shevell SK, Holliday I, Whittle P. Two separate neural mechanisms of brightness induction. Vision Res. 1992;32: 2331–2340. 1288009 10.1016/0042-6989(92)90096-2

[56] Sugita Y. Contrast and assimilation on different depth planes. Vision Res. 1995;35: 881–884. 7762145 10.1016/0042-6989(94)00217-a

[57] Festinger L, Coren S, Rivers G. The effect of attention on brightness contrast and assimilation. Am J Psychol. 1970;83: 189–207. 5450896

[58] Kingdom FAA. Lightness, brightness and transparency: A quarter century of new ideas, captivating demonstrations and unrelenting controversy. Vision Res. 2011;51: 652–673. 10.1016/j.visres.2010.09.012 20858514

[59] Fry GA, Alpern M. The effect of a peripheral glare source upon the apparent brightness of an object. J Opt Soc Am. 1953;43: 189–195. 13035557 10.1364/josa.43.000189

[60] Arend LE, Buehler JN, Lockhead GR. Difference information in brightness perception. Percept Psychophys. 1971;9: 367–370.

[61] McMahon MJ, Packer OS, Dacey DM. The classical receptive field surround of primate parasol ganglion cells is mediated primarily by a non-GABAergic pathway. J Neurosci; 2004;24: 3736–3745. 10.1523/JNEUROSCI.5252-03.2004 15084653 PMC6729348

[62] Leibowitz H, Mote FA, Thurlow WR. Simultaneous contrast as a function of separation between test and inducing fields. J Exp Psychol. 1953;46: 453–456. 13118076 10.1037/h0062595

[63] Fach C, Sharpe LT. Assimilative hue shifts in color gratings depend on bar width. Percept Psychophys. 1986;40: 412–418. 3808908 10.3758/bf03208201

[64] Rudd ME. Edge integration in achromatic color perception and the lightness–darkness asymmetry. J Vis. 2013;13: 1–30. 10.1167/13.14.18 24370541

[65] Zaidi Q, Yoshimi B, Flanigan N, Canova A. Lateral interactions within color mechanism in simultaneous induced contrast. Vision Res. 1992;32: 1695–1707. 1455741 10.1016/0042-6989(92)90162-c

[66] Blakeslee B, Pasieka W, McCourt ME. Oriented multiscale spatial filtering and contrast normalization: a parsimonious model of brightness induction in a continuum of stimuli including White, Howe and simultaneous brightness contrast. Vision Res. 2005;45: 607–615. 10.1016/j.visres.2004.09.027 15621178

[67] Land EH, McCann JJ. Lightness and retinex theory. J Opt Soc Am; 1971;61: 1–11. 5541571 10.1364/josa.61.000001

[68] Land EH. The retinex theory of color vision. Sci Am. 1977;237: 108–128. 929159 10.1038/scientificamerican1277-108

[69] Land EH. Recent advances in retinex theory and some implications for cortical computations: Color vision and the natural image. Proc Natl Acad Sci USA. 1983;80: 5163–5169. 6576382 10.1073/pnas.80.16.5163 PMC384211

[70] Provenzi E, Fierro M, Rizzi A, De Carli L, Gadia D, Marini D. Random spray Retinex: a new Retinex implementation to investigate the local properties of the model. IEEE Trans Image Process. 2007;16: 162–171. 17283775 10.1109/tip.2006.884946

[71] Bertalmío M, Caselles V, Provenzi E. Issues about retinex theory and contrast enhancement. Int J Comput Vis. 2009;83: 101–119.

[72] Rizzi A, Gatta C, Marini D. A new algorithm for unsupervised global and local color correction. Pattern Recognit Lett. 2003;24: 1663–1677.

[73] Bertalmío M, Cowan J. Implementing the Retinex algorithm with Wilson-Cowan Equations. J Physiol Paris. 2009;103: 69–72. 10.1016/j.jphysparis.2009.05.001 19477277

[74] Bertalmío M. From image processing to computational neuroscience: a neural model based on histogram equalization. Front Comput Neurosci; 2014;8: 1–9. 25100983 10.3389/fncom.2014.00071 PMC4102081

[75] Rudd ME. A cortical edge-integration model of object-based lightness computation that explains effects of spatial context and individual differences. Front Hum Neurosci; 2014;8: 1–14. 25202253 10.3389/fnhum.2014.00640 PMC4141467

[76] Rudd ME, Zemach IK. The highest luminance anchoring rule in achromatic color perception: some counterexamples and an alternative theory. J Vis. 2005;5: 983–1003. 10.1167/5.11.5 16441197

[77] Rudd ME, Zemach IK. Contrast polarity and edge integration in achromatic color perception. J Opt Soc Am A Opt Image Sci Vis. 2007;24: 2134–2156. 17621319 10.1364/josaa.24.002134

[78] Kamermans M, Kraaij D a, Spekreijse H. The cone/horizontal cell network: a possible site for color constancy. Vis Neurosci. 1998;15: 787–797. 9764521 10.1017/s0952523898154172

[79] Vanleeuwen MT, Joselevitch C, Fahrenfort I, Kamermans M. The contribution of the outer retina to color constancy: a general model for color constancy synthesized from primate and fish data. Vis Neurosci. 2007;24: 277–290. 10.1017/S0952523807070058 17592668

