# Supplementary material for: Retinal Lateral Inhibition Provides the Biological Basis of Long-Range Spatial Induction
Source: PLoS One. 2016 Dec 28;11(12):e0168963. doi: 10.1371/journal.pone.0168963 (PMC5193432; doi:10.1371/journal.pone.0168963)
Supplement: S1 Appendix — The details of the general simulation set ups on retinal projection, luminance unit, data acquisition and temporal spatial filtering methods are illustrated. (PDF) [file pone.0168963.s001.pdf]

S1 Appendix. Generic Simulation Methods

Retinal Projection

The visual field (i.e. the retinal area on which the stimulus was projected) was restricted to 8° for all model simulations. The stimuli were projected at the center of this projection area in sizes as specified in the behavioral studies. The initial values of the cells in each retinal layer were set to resting potential values (or, in an algorithmic term, steady state values of the equations) to the adapting light intensity (i.e. uniform illumination over the entire visual field) in each experiment.

We applied foveal photoreceptor density uniformly for the entire visual field (i.e. projection area) and disregarded the radial density reduction as a function of retinal eccentricity (fovea v.s. periphery). The foveal cell density was set to 146000 $photoreceptors/mm^2$ (111 x 111 cells per square degree) following Wilson's model [S1].

While our assumption on the cell density needs to be interpreted with a caution, we considered that disregarding the change of the spatial sampling rate across the retinal surface is rather compatible to the fact that, in reality, observers in the behavioral experiments [S2,S3] were not restricted from eye movements, in which case the observers would move their eyes to refocus different areas of the stimulus onto the fovea. This assumption on cell density is relatively unimportant for the simulations of Reid and Shapley's experiment in which the maximum size of the stimuli was 1.8º.

Luminance Unit

Both of the original models by Wilson [S1] and by van Hateren [S4,S5] are designed to take in troland (retinal illuminance) values. If the behavioral studies provided luminance values in cd/m^2 for stimulus intensity, the provided values were converted into troland unit.

As a standard, we set the pupil diameter to 3 mm, which is the value used in Wilson's model. Wilson defined the point-spread function of light scatter corresponding to the 3mm pupil based on Campbell and Gubisch [S6] and we kept all these aspects intact for all the simulations using Wilson's model.

We tested an additional pupil size with van Hateren's model (6.5 mm, from monocular viewing of 8 cd/m^2 field by a 30-year-old person based on the study by [S7]) and confirmed that the results in this study are unaffected by the assumption on pupil size. Noting that assuming a larger pupil size yields bigger troland values in retinal inputs than a smaller pupil size for the same luminance values in cd/m^2, this means that our results are robust over different retinal illumination range.

Data Acquisition

Since the retinal models compute voltage responses that change across time, it is rather arbitrary to set a particular point in time in which the cell responses correspond to a perceptual experience. Given the difficulty, we have chosen the time point for data acquisition to be at 300 ms after stimulus onset (assuming that a stimulus did not set off before 300 ms). This time scale allows all the short-term feedback effect (i.e. the inhibitory feedback from horizontal and amacrine cells in Wilson's model; immediate feedback from horizontal cell without the effect of adaptive gain in van Hateren's model) to be stabilized, while this duration is insufficient for the long-term adaptive system to take significant effect (i.e. interplexiform layer cell feedback to horizontal cells for gain and temporal change in Wilson's model; adaptive horizontal gain change in van Hateren's model). Thus, all the data reported in the current study are derived by analyzing the model cell responses at 300 ms after the stimulus onset.

Temporal and Spatial Filtering

For all simulations, the solution of a first-order ordinary differential equation (ODE) for temporal low-pass filtering was approximated by the modified Tustin method [S8], which is shown to outperform other ODE approximation schemes in terms of computational accuracy and speed among autoregressive moving-average filtering methods. The time step of the temporal evolution was set to 0.1 ms.

The spatial filtering was performed by frequency-domain convolution. As explained in the previous sections (The Model by Wilson and The Model by van Hateren), the spatial filters were generated in the functional forms described in each of the original papers (Gaussian point-spread function in Wilson's model; weighted sum of a wide and a narrow exponential point-spread functions in van Hateren's model, 2007).

References

S1. Wilson HR. A neural model of foveal light adaptation and afterimage formation. Vis Neurosci. 1997;14: 403–423.

S2. Helson H. Studies of anomalous contrast and assimilation. J Opt Soc Am. 1963;53: 179–184.

S3. Reid RC, Shapley R. Brightness induction by local contrast and the spatial dependence of assimilation. Vision Res. 1988;28: 115–32.

S4. van Hateren JH. A cellular and molecular model of response kinetics and adaptation in primate cones and horizontal cells. J Vis. 2005;5: 331–347.

S5. van Hateren JH. A model of spatiotemporal signal processing by primate cones and horizontal cells. J Vis. 2007;7: 1–19.

S6. Campbell FW, Gubisch RW. Optical quality of the human eye. J Physiol. 1966;186: 558–578.

S7. Watson AB, Yellott JI. A unified formula for light-adapted pupil size. J Vis. 2012;12: 1–16.

S8. van Hateren JH. Fast recursive filters for simulating Nonlinear dynamic systems. Neural Comput. 2008;20: 1821–1846.
